# How does Twitter account moderation work? Dynamics of account creation and suspension on Twitter during major geopolitical events

**DOI:** 10.1140/epjds/s13688-023-00420-7

**Published:** 2023-10-04

**Authors:** Francesco Pierri, Luca Luceri, Emily Chen, Emilio Ferrara

**Affiliations:** 1 Information Sciences Institute, University of Southern California, Los Angeles, USA; 2 Dipartimento di Elettronica, Informazione e Bioingegneria, Politecnico di Milano, Milano, Italy; 3 Department of Innovative Technologies, University of Applied Sciences and Arts of Southern Switzerland, Lugano, Switzerland; 4 Thomas Lord Department of Computer Science, University of Southern California, Los Angeles, USA; 5 Annenberg School of Communication and Journalism, University of Southern California, Los Angeles, USA

**Keywords:** Crisis, Moderation, Platform abuse, Social media, Twitter

## Abstract

Social media moderation policies are often at the center of public debate, and their implementation and enactment are sometimes surrounded by a veil of mystery. Unsurprisingly, due to limited platform transparency and data access, relatively little research has been devoted to characterizing moderation dynamics, especially in the context of controversial events and the platform activity associated with them. Here, we study the dynamics of account creation and suspension on Twitter during two global political events: Russia’s invasion of Ukraine and the 2022 French Presidential election. Leveraging a large-scale dataset of 270M tweets shared by 16M users in multiple languages over several months, we identify peaks of suspicious account creation and suspension, and we characterize behaviors that more frequently lead to account suspension. We show how large numbers of accounts get suspended within days of their creation. Suspended accounts tend to mostly interact with legitimate users, as opposed to other suspicious accounts, making unwarranted and excessive use of reply and mention features, and sharing large amounts of spam and harmful content. While we are only able to speculate about the specific causes leading to a given account suspension, our findings contribute to shedding light on patterns of platform abuse and subsequent moderation during major events.

## Introduction

Social media play a major role in modern democracies, enabling individuals to openly discuss political and societal issues as well as respond to crises and emergencies [1, 2]. However, they also expose users to a variety of harmful content that is often promoted by malicious actors in a coordinated fashion [3–7]. In recent times, we witnessed a rise of hate speech, conspiracy, and disinformation campaigns [8–10]. An “infodemic” of misleading and inaccurate information became particularly worrisome during the COVID-19 pandemic [11–18]. Social platforms traditionally take steps to mitigate damage by moderating content and by de-platforming, i.e., removing or suspending accounts that engage in harmful activity [17, 19, 20]. Numerous high-profile cases of influential individuals fueling conspiracy theories and inciting violence [21] have led to real-world incidents and subsequent public outcry [22], bringing platforms to intervene by deactivating accounts of public figures like Donald Trump and Alex Jones.^1^ But moderation has sparked a vivid debate among academics, journalists, and policy-makers, as it might pose threats to freedom of speech [23]. A shift toward soft-moderation approaches, which include down-ranking (i.e., lowering the visibility of certain content in other users’ feeds), “shadow banning” (i.e., not showing that content to other users), and warning labels (i.e., tagging content as potentially harmful or inaccurate), has been recently noticed within platforms’ moderation tactics [24, 25].

To further our understanding of how these moderation strategies are enacted to tame abuse, here we study Twitter accounts’ creation and suspension dynamics. Existing research into suspended accounts on Twitter spans various settings, from political elections to social movements [26–28]: a limitation to these studies is that their analyses are retrospective, i.e., done by querying Twitter’s Application Programming Interface (API) with a considerable delay (sometimes years) with respect to the period of activity of observed users, hence preventing the determination of when and why accounts were actually suspended, information that are not disclosed by the platform. As labeled ground truth data about legitimate and abusive accounts is not always available, researchers typically rely on labeled datasets that emerged during audits or investigations into these platforms [29]. One such example is the case of the Russian *Internet Research Agency* state-controlled accounts [30], whose Twitter handles were released by the US Congress; however, Twitter never disclosed how they identified such malicious actors. This lack of transparency can hinder our understanding of moderation dynamics and perhaps the generalizability of associated findings. We attempt to address these two problems (retrospective labeling and opaque labels) in this study by timely tracking newly created accounts, and identifying suspended users with minimum delay with respect to the period of observation, with the goal of ascertaining whether and when they were suspended by Twitter. Since monitoring the whole platform would be operationally unfeasible, due to the API’s limitations, we focus on two major events that sparked significant attention and are conducive to controversy, e.g., geopolitical events [31, 32], hence becoming fertile ground for platform abuse, namely the 2022 Russia-Ukraine war and the 2022 French Presidential election.

The choice of these two events is justified by documented evidence of platform abuse and subsequent Twitter interventions. For example, after the invasion of Ukraine, researchers and journalists raised attention to a suspicious spike in accounts created on Twitter that were engaging in conversations around the war [33, 34]. Noticing that many of these accounts were being immediately suspended, it was suggested that the accounts were most likely responsible for the coordinated spread of Russian propaganda. In addition to the geopolitical context provided by the Russo-Ukrainian conflict, we considered a political scenario by focusing on the 2022 French Presidential election, since the previous election in 2017 attracted a considerable amount of malicious activity and coordinated disinformation campaigns [35].

### Research questions

Based on these premises, we aim to explore the dynamics of account creation and suspension during the two different major events by collecting information about suspension in a timely fashion. Specifically, we address the following research questions:

- **RQ1**: What are the temporal dynamics of account creation (*RQ1a*) and suspension (*RQ1b*) around major events?
- **RQ2**: Do new and suspended accounts exhibit different behaviours compared to active users (*RQ2a*)? What are their patterns of interactions (*RQ2b*)?
- **RQ3**: What kind of content do suspended accounts share?

We collected and analyzed over 270M tweets in multiple languages, to show that the increase in activity on Twitter during the two major geopolitical events is accompanied by peaks in account creation and abusive behavior, exposing legitimate users to spam campaigns and harmful speech. In particular, we found that Twitter tends to be more proactive towards suspending newly created user accounts, compared to older existing accounts. We also highlight several behavioral features that differentiate users who get suspended from regular and active users, finding very similar results across the two scenarios, and providing insights for research that aims to better understand platforms’ policies to handle digital misbehavior and online abuse.

## Related work

In this section, we first review existing contributions focusing on suspended users on Twitter, then we provide an overview of work related to the ongoing conflict in Ukraine and the French Presidential elections.

### Suspended accounts

Early work on abusive usage of social platforms, which leads to account suspension and removal, mostly focuses on detecting spam, bots, and state-backed trolls [36–42]. Recently, several contributions carried out retrospective analyses of suspended and deactivated accounts on Twitter in various contexts.

Le et al. [26] provide a “postmortem” analysis of approximately 1M accounts that were active during the 2016 US Presidential election, showing different classes of tweeting behaviors and identifying different communities.

Following a similar approach, Chowdhury et al. [43] identify over 2M suspended accounts that engage mostly in political and marketing campaigns, showing that over 60% of them were active for more than two years on Twitter before being suspended. In follow-up work, the same authors [44] aim to identify factors that lead to suspension during the 2020 US Presidential election, showing that suspended users use more curse and derogatory words, and tend to share more right-leaning news.

Toraman et al. [27] focus on approximately 500k suspended and deleted users who engaged with the “Black Lives Matter” social movement, characterizing their behavior in terms of spam, negative language, hate speech, and misinformation spread.

Seyler et al. [45] tackle the problem of automatically identifying and predicting users’ suspension, leveraging tweeting behavior and linguistic cues in the messages shared by suspended and regular users. Leveraging deep neural networks, they achieve up to 82% accuracy in the binary task of classifying users as suspended or not.

Lastly, Majo et al. [28] carry out a multi-country analysis of users who got suspended during political elections in 2017 in multiple countries (France, Germany, and the United Kingdom). They show how the behavior and content shared by Twitter suspended accounts are significantly different compared to other active accounts, as they focused more on amplifying divisive issues like immigration and religion.

As we detail next, the present work aims to overcome the main limitation of the above contributions, namely the great amount of delay in the identification and analysis of suspended users with respect to the data collection. In contrast to previous approaches that studied suspension patterns years after the targeted event, our approach focuses on the analysis of suspended users within weeks. While we explore different dimensions and characteristics of Twitter users compared to the aforementioned analyses, we do report findings that align with such previous results.

### 2022 Russia-Ukraine war and French election

Following the Russian invasion of Ukraine, the Observatory on Social Media at Indiana University investigated the prevalence of suspicious activity on Twitter. In a series of white papers [33, 34], they highlight a peak in the creation of new accounts around the day of the invasion, and reveal the presence of coordinated groups of users promoting different campaigns, from boosting the presence of political figures to spam and hate speech. They also show how most of the related messages shared on Twitter are genuine or benign, and that pro-Ukraine messages are much more prevalent than pro-Russia ones.

Caprolu et al. [46] apply mix-methods to analyze a collection of over 5M tweets related to the ongoing conflict, claiming no evidence of massive disinformation campaigns contrary to what was reported in the mainstream news. Park et al. [47] introduce a dataset called VoynaSlov, which aims to help researchers study Russian language conversations on Twitter and VKontakte (VK), a social platform very popular in Russia, focusing on the attention received by state-affiliated and independent Russian media.

Hanley et al. [48] leverage a sentence-level topic analysis technique to study the spread of Russian state propaganda on Reddit, by analyzing the content generated by Russian disinformation websites from January to April 2022. They find approximately 40% of the comments in the *r/Russia* subreddit are related to Russian disinformation. The same authors [49] use a combination of sentiment and topic analysis to study Western, Russian, and Chinese media on Twitter and Weibo, finding that Russian media attempt to justify their “special military operation” as opposed to Western press that mostly covers military and humanitarian aspects of the war. Chinese news, instead, insists on the conflict’s diplomatic and economic backlashes.

Geisller et al. [50] study the activity of automated accounts promoting pro- and anti-Russia hashtags on Twitter, in order to quantify the extent to which social bots might influence human accounts by promoting and amplifying Russian propaganda and disinformation.

Pierri et al. [51] study the spread of Russian propaganda and misinformation on Facebook and Twitter during the first months of the conflict. They estimate the prevalence of such content on the two platforms, describing temporal patterns and highlighting the disproportionate role played by superspreader accounts. They also estimate the amount of content removed by the two platforms to be around only about 8-15% of the posts and tweets sharing links to untrustworthy sources.

There is a substantial corpus of literature discussing platform abuse and manipulation on Twitter during political elections in different countries [5, 52–55]. For what concerns French Presidential elections, early work by Ferrara [35] studies the presence of disinformation and the role of bots prior to the 2017 election, with a focus on the *MacronLeaks* campaign. They show that users who engaged the most with the campaign were mostly non-French users active in promoting fringe and alt-right narratives, suggesting the possible existence of a black market for reusable political disinformation bots.

Recently, Abdine et al. [56] analyze the 2022 French election by showing that supporters of certain candidates engage more in hate speech and aggressive behavior, and revealing the presence of likely bot activity.

## Data collection

We employed two different Twitter datasets for our analyses, both collected through the Standard v1.1 Streaming endpoint.^2^

The first dataset [57] contains tweets matching keywords related to Russia’s invasion of Ukraine—which occurred on February 24th, 2022—in the period from February 22nd to April 28th, 2022. Over 30 keywords in English, Russian and Ukrainian languages were identified by looking at trending topics and hashtags. We refer the interested reader to the related publication [57] for more details on the collection procedure. The data comprises 230,166,962 tweets shared by 14,995,636 unique users. A sample of keywords is available in Table 1, whereas the full list is available in the repository associated with the dataset,^3^ which also contains IDs of tweets that can be re-hydrated querying the Twitter API or using tools like *Hydrator*^4^ or *twarc*.^5^ We will refer to this dataset as UK-RU. We refer the reader to a similar dataset [58] which is available at https://github.com/Leibniz-HBI/ukraine_twitter_data.

**Table 1:** Sample of keywords employed to collect tweets relevant to Russia’s invasion of Ukraine

ukraine	russia	Putin	SlavaUkraini	ukrainian
soviet	kremlin	nato	kyev	moscow
zelensky	fsb	kgb	donbas	luhansk

The second dataset contains tweets related to the 2022 French Presidential election—held on April 10th and April 24th, respectively—in the period from April 3rd to May 15th 2022. We employed a snowball sampling approach [59] at the end of March to identify 89 relevant keywords in English and French language. The data comprises 39,724,541 tweets shared by 2,792,499 unique users. A sample of keywords is available in Table 2, whereas the full list is available in the repository associated with the dataset.^6^ We will refer to this dataset as FR-22.

**Table 2:** Sample of keywords employed to collect tweets relevant to the 2022 French Presidential elections

macron	lepen	zemmour
zemmourtrocadero	mckinseygate	mckinseymacrongate
scandalemacron	zemmourbfm	reconquete2022

**Figure 1:**
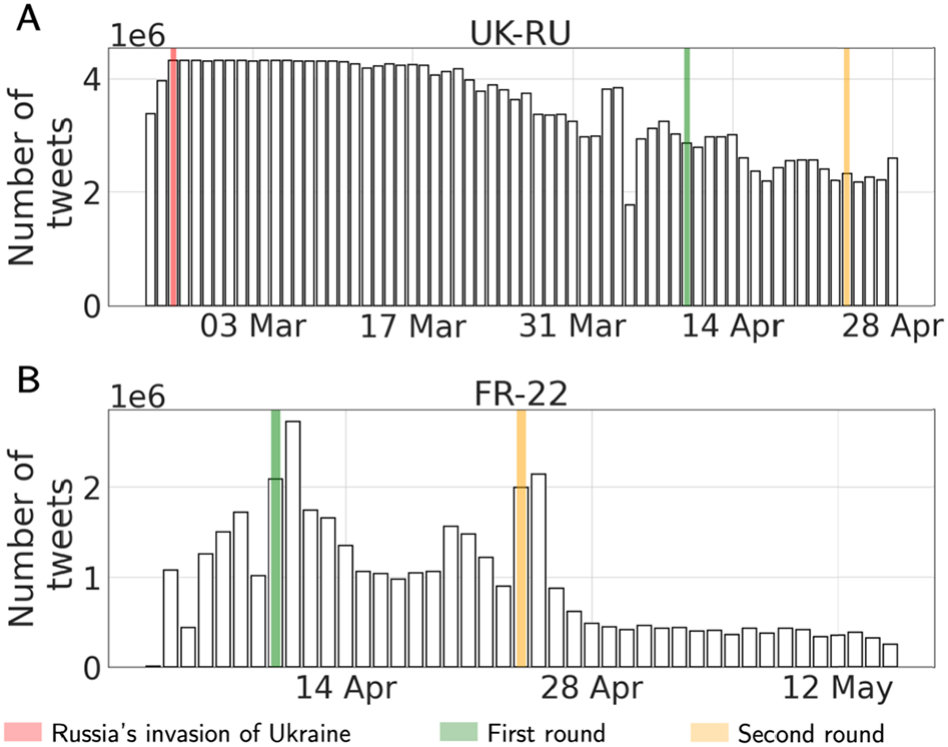
Time series of the daily number of tweets collected in UK-RU **(left)** and FR-22 **(right)** datasets. Notice the drop in volume on April 7th for UK-RU, which corresponds to a network malfunctioning failure lasting a few hours. N.B: the x-axes are not aligned in the two subplots

A limitation in this data collection strategy is the 1% maximum sampling-rate imposed by Twitter on the streaming endpoint [60]. The issue of hitting the maximum rate limit occurred occasionally in the case of UK-RU, as it can be seen in the left panel of Fig. 1, where the data volume saturates at around 4M daily tweets during the first week of the invasion; it did not arise for FR-22 (see the right panel in Fig. 1).

We further identified suspended accounts by leveraging the POST/2/compliance/jobs endpoint via twarc2.^7^ Specifically, on May 23rd we queried Twitter for all the accounts that shared a tweet in UK-RU and FR-22, obtaining almost 2M users that were suspended by the platform for violating their rules. Twitter might suspend an account in a variety of circumstances that range from promoting violence and glorifying crime to hate speech, spam, and impersonation; similarly to other Big Tech platforms, these guidelines are considered among the most stringent [61]. More details about reasons for suspension are available in the Twitter documentation.^8^

## Results

In the following sections, we provide answers to our research questions from multiple angles. We first look into patterns of account creation and suspension in both datasets. We then analyze the behavioral features and interactions that characterize different classes of accounts. Finally, we describe the type of content shared by suspended users in contrast with legitimate active accounts.

### Patterns of account creation (RQ1a)

**Figure 2:**
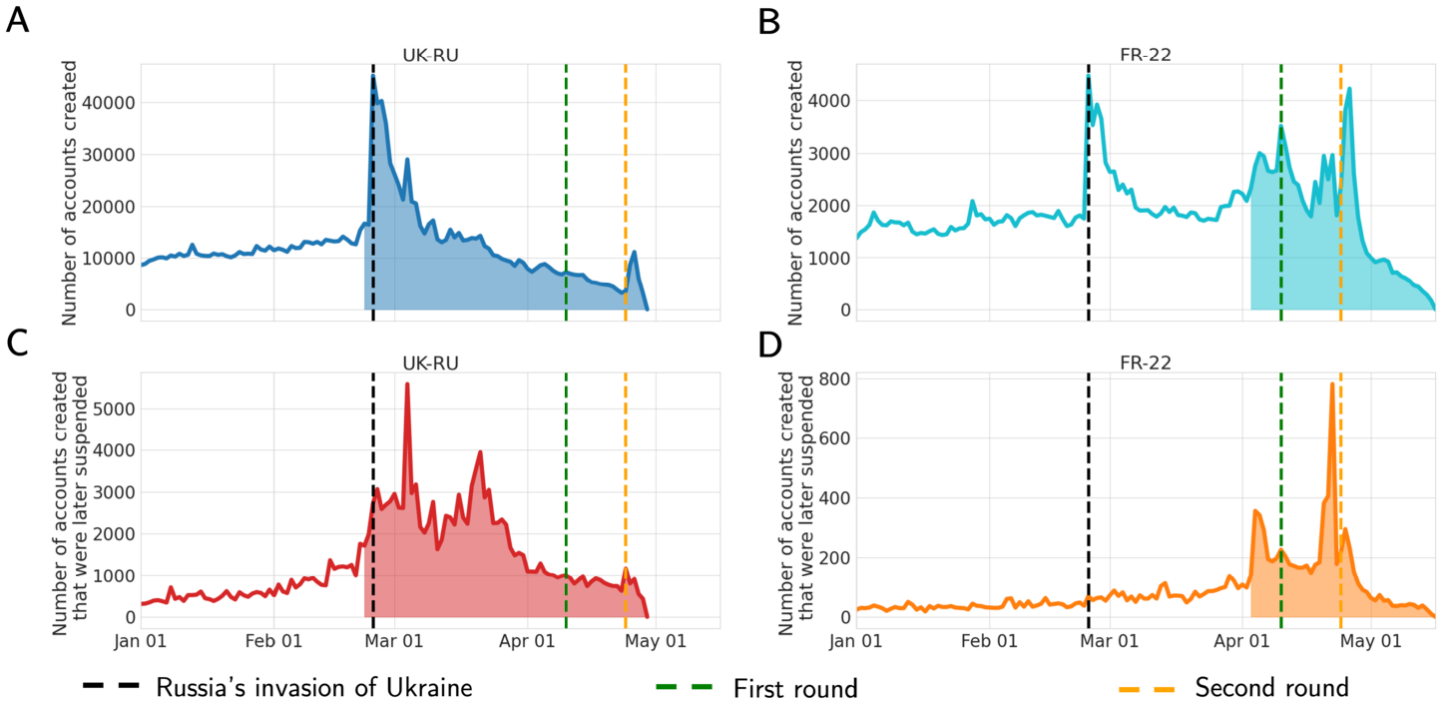
Time series of the daily number of accounts created in UK-RU **(A)** and FR-22 **(B)** since January 1st to May 15th 2022. Time series of the daily number of accounts created and that were later suspended (as of May 23rd) in UK-RU **(C)** and FR-22 **(D)**. Colored areas indicate the actual collection period in each dataset. The creation date of accounts is available in the Twitter user object provided by the API

Panels **A** and **B** in Fig. 2 show the daily number of accounts created in UK-RU and FR-22, respectively. We obtained information about account creation from the user object provided by Twitter API, and we are therefore able to count the number of users that were created before the data collection period. In line with [33, 34], we notice a peak of accounts created in correspondence with the Russian invasion of Ukraine (February 24th) in UK-RU, followed by a consistent decrease over time. A similar increase is observed in FR-22, and additional peaks are observed in correspondence of the two rounds of elections in FR-22. We also notice a significant peak of account creations in both datasets around April 26th, when Elon Musk announced the deal to buy Twitter. An investigation into this uniquely peculiar peak will be tackled in a separate study.

A total number of 863,017 accounts (5.75% of all active users in the dataset) were created during the collection period in UK-RU, whereas 80,623 accounts (2.88% of all active users) were created in FR-22. The proportion in the former dataset is larger, most likely because the conflict captivated a larger audience on a global scale, and because the collection period was slightly longer.

Panels **C** and **D** in Fig. 2 show the number of accounts created on each day that were later suspended (as of May 23rd): for UK-RU, 121,548 accounts out of 288,723 suspended accounts (∼42.1%), whereas for FR-22 7073 accounts out of 24,805 suspended accounts (∼28.5%) were created during this period.

We can notice two different dynamics: in UK-RU, we observe an increase of suspicious accounts created after the invasion, with a few peaks in March followed by a decreasing trend; in FR-22 we observe two peaks of creation that precede the two rounds by a few days, which suggest the possible implementation of strategies to pollute online conversations and/or promote specific narratives before the voting events. Besides, the proportion of new accounts that get suspended is larger in UK-RU (14%) than in FR-22 (8.77%). Similarly, the proportion of suspended accounts that are created during the period of observation is much larger in UK-RU (42.1%) than in FR-22 (28.5%).

One possible explanation could be that the massive audience engaging in online conversations around the conflict consequently lured in a larger amount of malicious activity compared to the French election. These findings confirm previous work which showed peaks of suspensions [26, 43] and a larger number of new accounts among those suspended [26, 28, 44].

### Patterns of account suspension (RQ1b)

As Twitter’s API does not provide information about the specific time when an account gets suspended, we consider the last appearance of a suspended account as a proxy for suspension time, i.e., the last tweet authored by or targeting (mentioning/replying/retweeting/quoting) the account.

**Figure 3:**
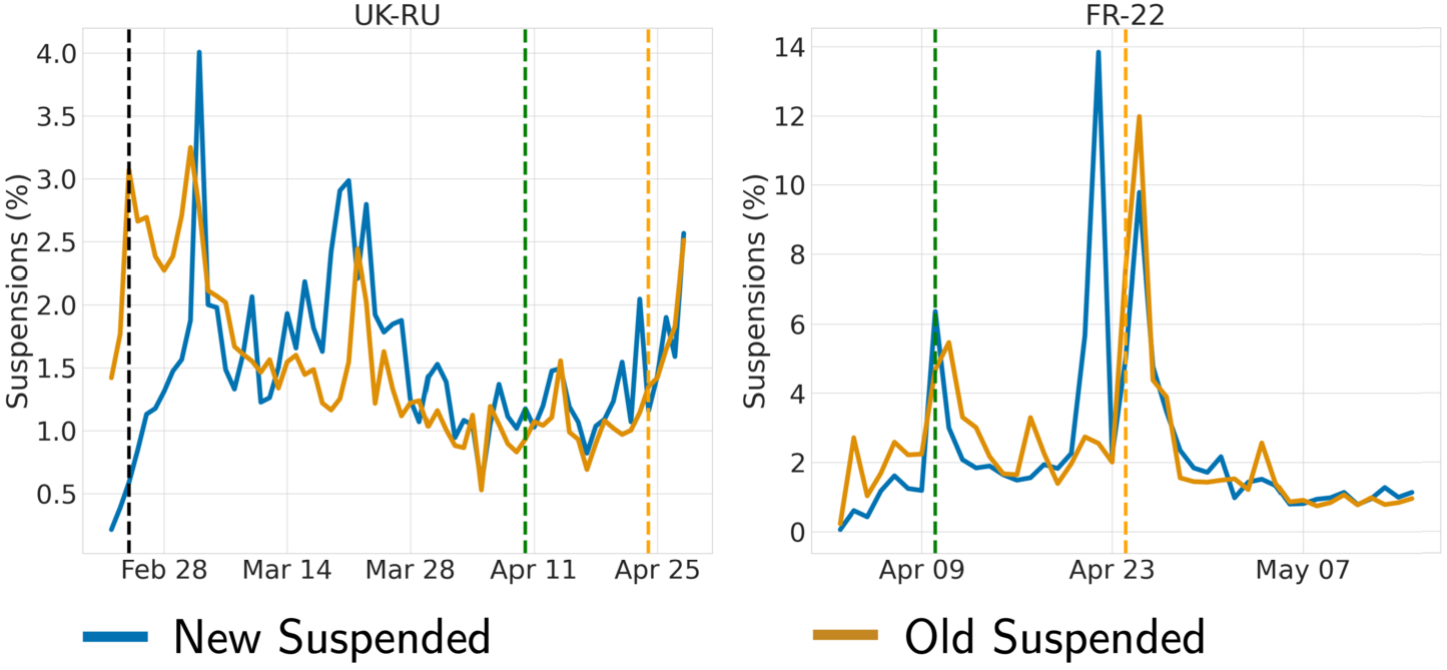
Time series of the daily proportion of accounts that were suspended in UK-RU **(left)** and FR-22 **(right)** out of all suspended accounts in each dataset, separating accounts created during the collection period (New) from those already existing (Old). Vertical lines indicate the invasion of Ukraine (black) and the two rounds of elections (green and light orange). N.B: the x-axes are not aligned in the two subplots

Figure 3 shows the daily proportion of accounts that got suspended out of all suspended accounts in each dataset, by labeling accounts created during the collection period as *New*, as opposed to *Old* accounts who were already present on the platform at collection time. We can notice slightly different patterns of suspension between the two groups in UK-RU (Pearson $R=0.297$, $p = 0.015$), whereas there is a stronger correlation in FR-22 (Pearson $R=0.628$, $p<0.001$).

Overall, we can notice a few peaks of suspension events for *New* accounts in UK-RU that correspond to peaks in account creation altogether (cf., Fig. 2), suggesting that a large proportion of those accounts were probably created to deceive, manipulate or pollute online conversations. On the other hand, we can observe peaks of suspension in correspondence of the election rounds in FR-22, suggesting a higher level of awareness by the platform with the aim of preserving the integrity of conversations on the election days (cf., the peak of suspension of new accounts a few days before the 2nd round). This result is in line with previous work [26, 43].

We further investigate the lifespan of users who get suspended, both in *relative terms* (i.e., the number of days since their first appearance in the dataset to their suspension), and in *absolute terms* (i.e., the number of days since their creation, for *New* accounts only).

**Figure 4:**
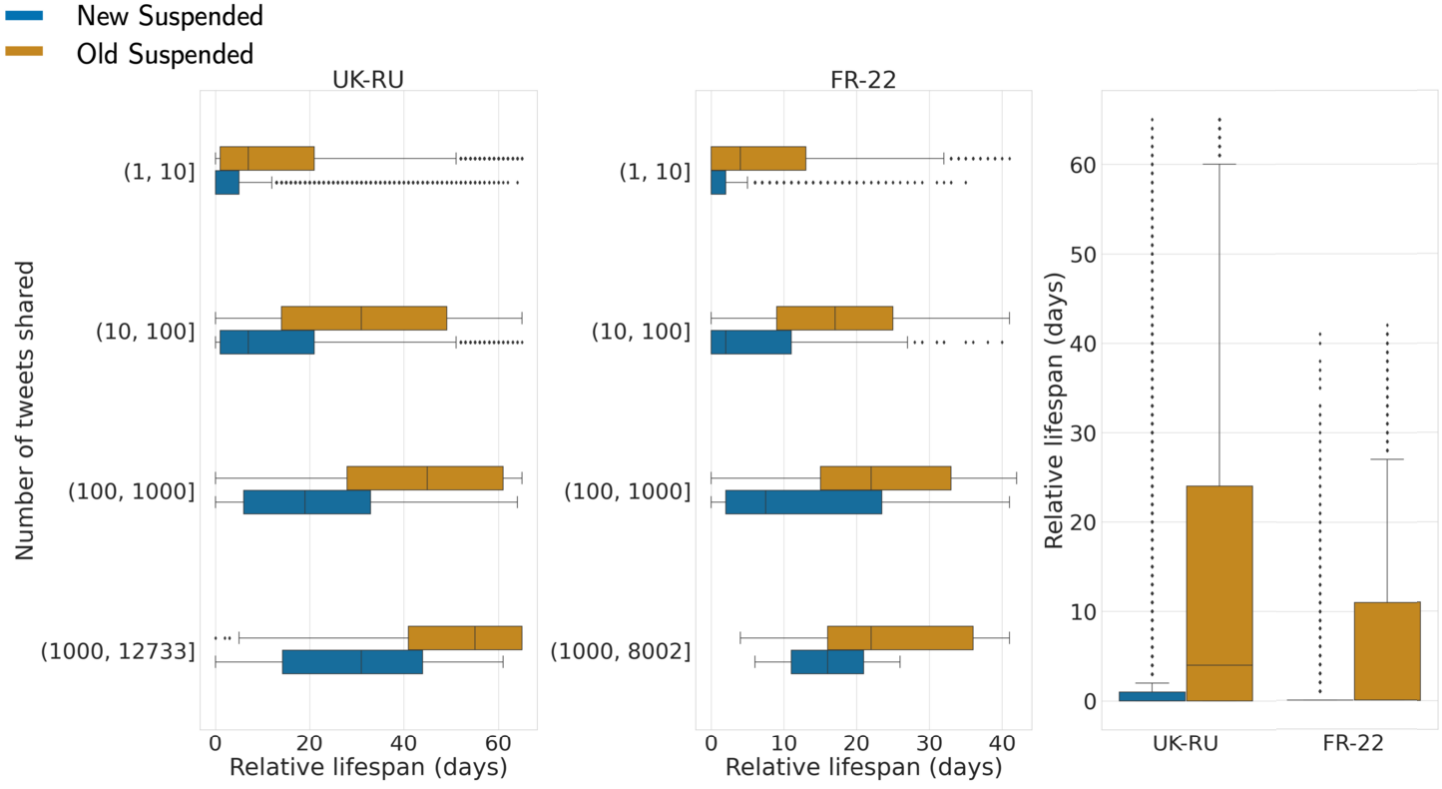
Distribution of relative lifespan matching users on the number of tweets shared in the two datasets, for UK-RU **(left)** and FR-22 **(center)**. For UK-RU the median values are 0 days and 4 days, respectively for *New* and *Old* suspended users; for FR-22 it is 0 days for both *New* and *Old* suspended users. Distribution of the relative lifespan of *New* and *Old* suspended users in both datasets **(right)**. All distributions of the two classes of users are statistically different according to two-sided Mann-Whitney tests ($p < 0.001$)

**Figure 5:**
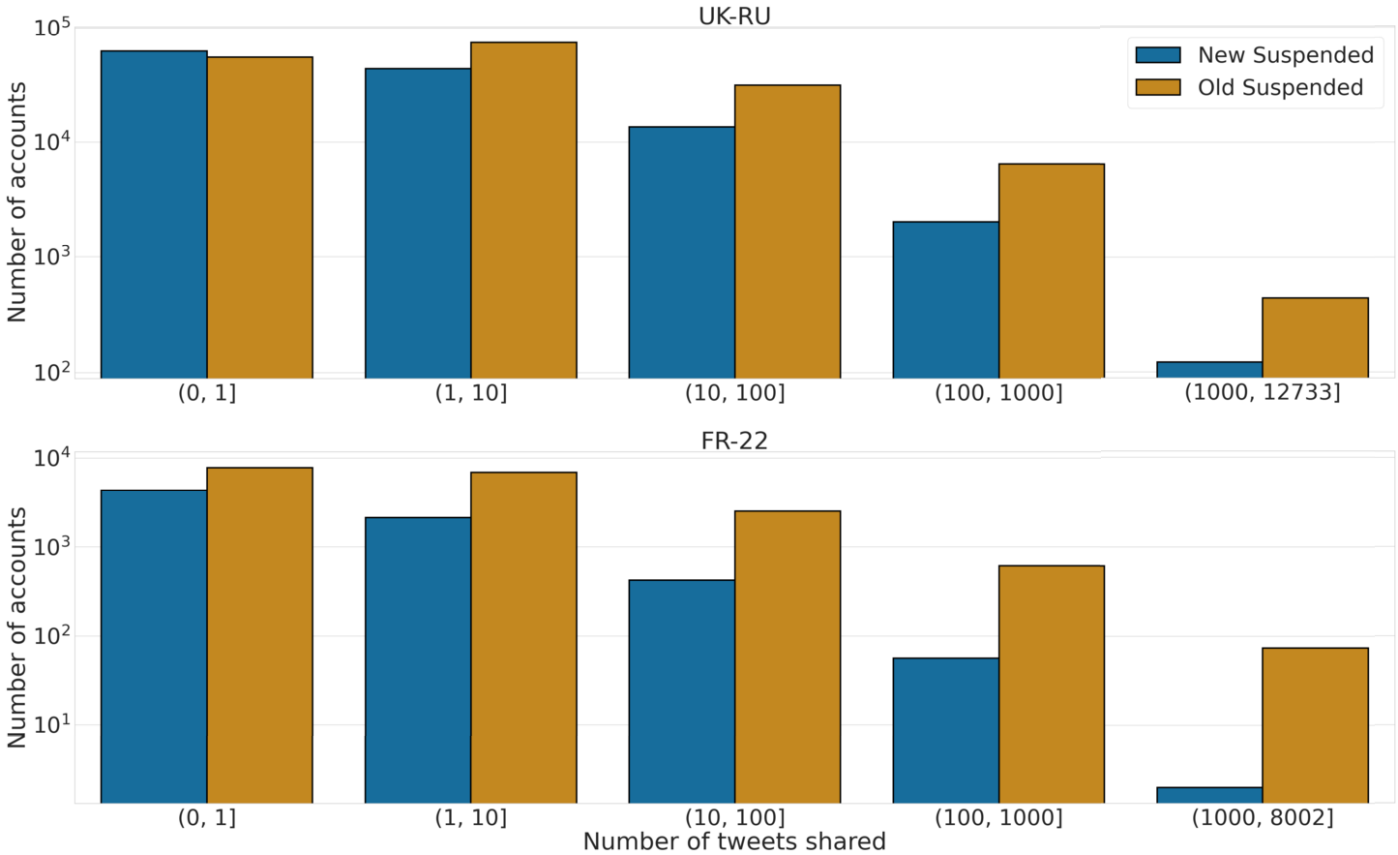
Distribution of the number of New and Old Suspended users for UK-RU **(top)** and FR-22 **(bottom)**, binned by the number of tweets shared

Panel **A** in Fig. 4 shows the distribution of the relative suspension time for both datasets; we observe that *New* accounts get suspended significantly earlier than *Old* ones in both cases. Given that most users shared only a handful of tweets (e.g., 20% of *New* and *Old* suspended accounts shared only 1 tweet in our datasets), we investigate users’ lifespan based on their sharing activity. We perform an exact matching of users based on their number of shared tweets in the two datasets, using the following logarithmic bins: $(1, 10]$, $(10, 100]$, $(100, 1000]$ and $(1000, M]$, where *M* is the maximum number of tweets shared by a suspended user in each dataset, respectively $M_{{\mathtt{UK}\text{-}\mathtt{RU}}} = 12{,}733$ and $M_{{\mathtt{FR}\text{-}\mathtt{22}}} = 8002$. We show in Fig. 5 the number of users present in each bin, for each dataset. We can notice that approximately over 50% of the accounts shared less than 10 tweets, and that hyper-active users (those with more than 100 tweets shared) are much more prevalent in Old Suspended accounts than in New Suspended accounts, in both datasets.

Panel **B** in Fig. 4 shows the distributions of relative lifespan for both classes of suspended users in UK-RU, and similar results are shown in panel **C** for FR-22; note that we only consider accounts that shared more than 1 tweet. We observe that *New* users get suspended earlier than *Old* users regardless of their tweeting activity, but we also notice that more active users are generally suspended later, accentuating the discrepancy between the two groups. This suggests that the age of an account might be a feature considered by Twitter to promptly detect suspicious accounts at scale, in accordance with previous literature [27, 28, 43].

**Figure 6:**
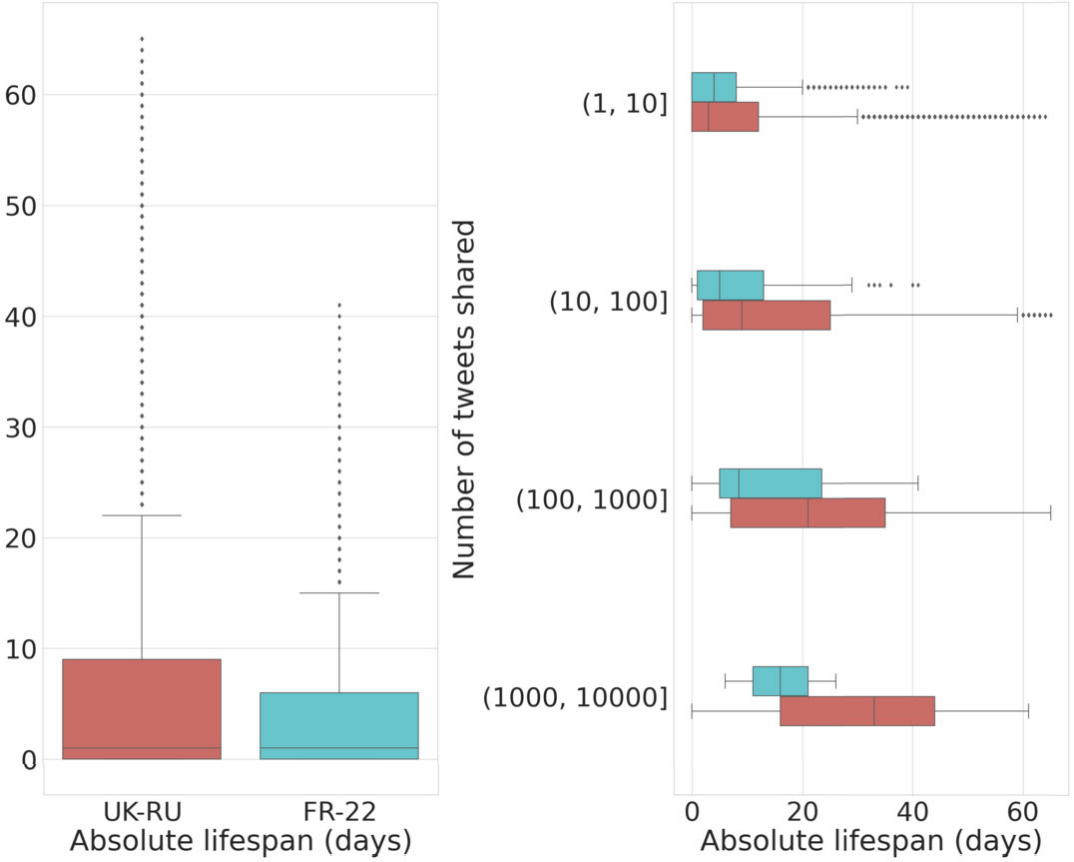
Distribution of the absolute lifespan of *New* suspended users **(left)** in both datasets (median value is 1 day for both datasets). Same distribution but matching accounts on the number of tweets shared **(right)**

We observe similar results for new users when considering their absolute lifespan (cf. left panel in Fig. 6, the median lifespan is 1 day in both datasets). In particular, we notice that users in FR-22 were suspended earlier than their counterparts in UK-RU, especially those very active (see right panel of the Figure): this might be attributed to the fact that the timescale of the French election event was significantly shorter than that of the Russia-Ukraine conflict and that the event was not so unexpected as the Russian invasion, hence Twitter therein enacted more prompt account suspensions.

We caution that our analysis presents a few caveats, as we only process tweets shared by users in a specific context (dataset) and, thus, we might not be observing the reasons that lead to each given account’s suspension. Also, given the limited period of analysis, we do not have the same amount of information for each *New* account, i.e., we collect fewer observations for those created toward the end of the collection period compared to those created at the beginning, and the length of the collection period differs for the two datasets.

### Behaviour of suspended accounts (RQ2a)

We first defined two additional classes to investigate whether suspended users exhibit a different behaviour compared to active users—*New* and *Old* active accounts—depending on their creation date. These classes are highly imbalanced as most users belong to the *Old Active* class (93% in UK-RU and 96% in FR-22). For each group of users, we computed the following sets of features:

- Proportion of original tweets, replies, retweets, and quotes out of all their shared tweets.
- Initial and final number of followers.
- Proportion of *contextual* tweets, i.e., the ratio between the number of tweets shared in the dataset and the final statuses count in their profile, i.e., the total number of tweets shared by the user on Twitter.
- Mean inter-tweeting time, i.e., the average time (in seconds) between two consecutive tweets shared by a user.

The last feature can be seen as a lower bound of the relative activity of a user in a particular context (dataset)—despite known limitations derived from sampling biases and deletion activity [62].

**Figure 7:**
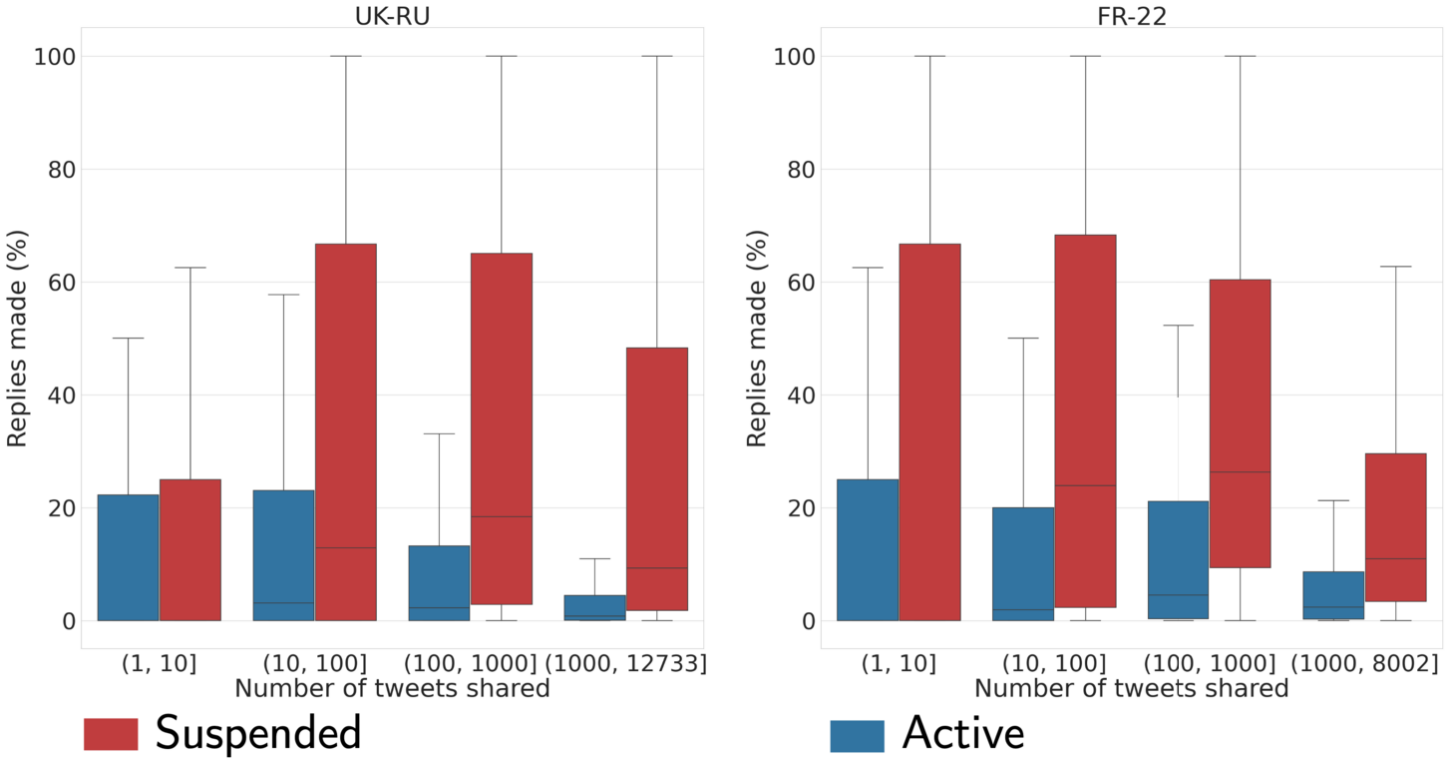
Distribution of the proportion of tweets that are replies for *Active* and *Suspended* accounts in UK-RU **(left)** and FR-22 **(right)**. Boxplots do not show outliers

We first compared *Suspended* versus *Active* users, by looking at the distributions of the aforementioned features with an exact matching procedure based on the number of tweets. We observe in both datasets that *Suspended* accounts made a significantly larger usage of replies compared to *Active* users in both datasets (see Fig. 7). Accordingly, *Suspended* accounts retweeted less and shared fewer original tweets/quotes w.r.t. *Active* users. This difference holds also when considering *Old* or *New* users separately, and in both datasets.

**Figure 8:**
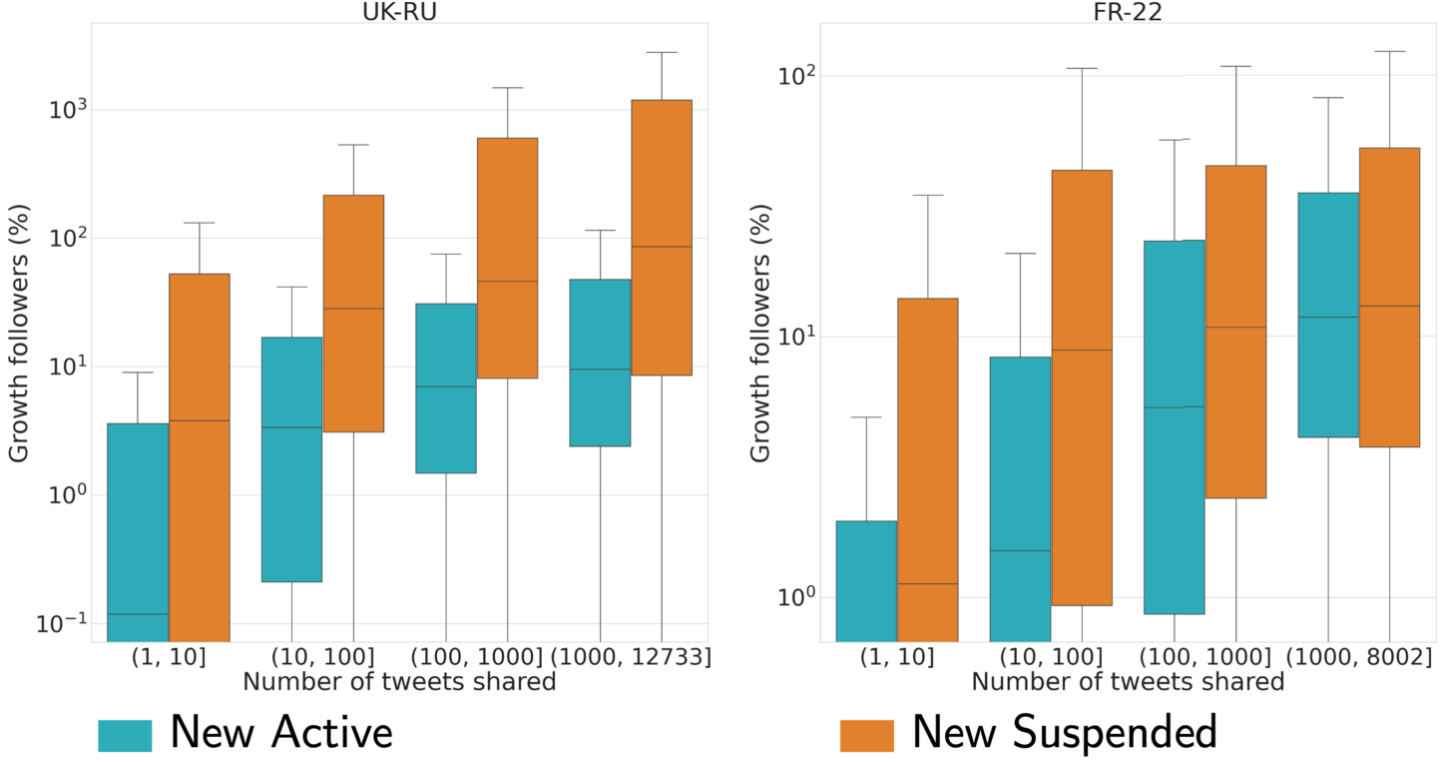
Distribution of the proportion of growth in followers (in percentage) for *New Active* and *New Suspended* users in UK-RU **(left)** and FR-22 **(right)**. Boxplots do not show outliers. The y-scale is logarithmic

We then looked at the differences between *New Suspended* and *New Active* users, and we observe that the latter group exhibits a larger growth in followers (i.e., the final minus initial number of followers) in both datasets but more visible in UK-RU, also when controlling for lifespan (see Fig. 8).

**Figure 9:**
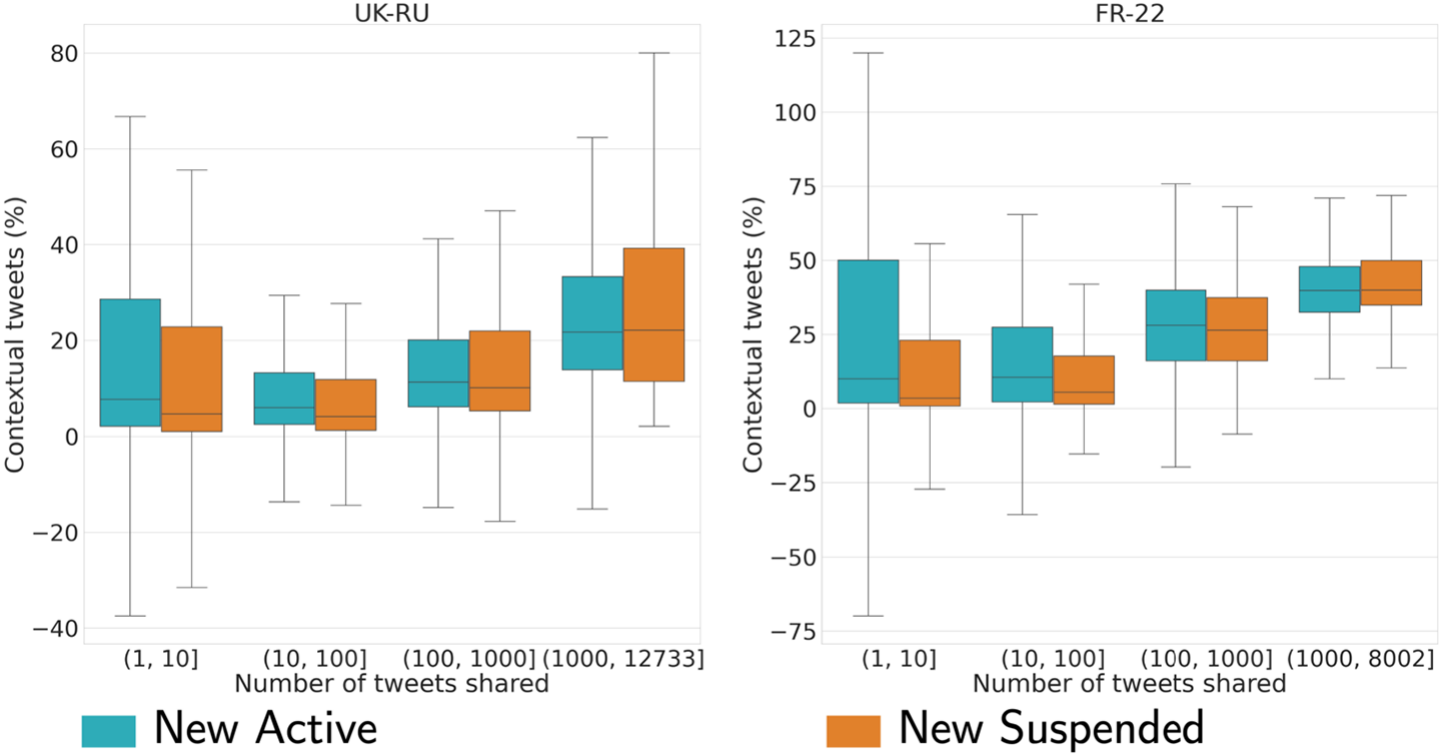
Distribution of the proportion of contextual tweets for *New Active* and *New Suspended* users in UK-RU **(left)** and FR-22 **(right)**. Boxplots do not show outliers. Negative values are due to accounts that deleted tweets during the period of observation, thus the difference between the final and the initial number of statutes is negative

We also considered the proportion of contextual tweets, which is similar among the two classes, and observe that it increases with the tweeting activity of users, although the median value stays below 50% in both datasets (see Fig. 9). This is because accounts partake to some extent in other discussion topics not captured by our data collection.

**Figure 10:**
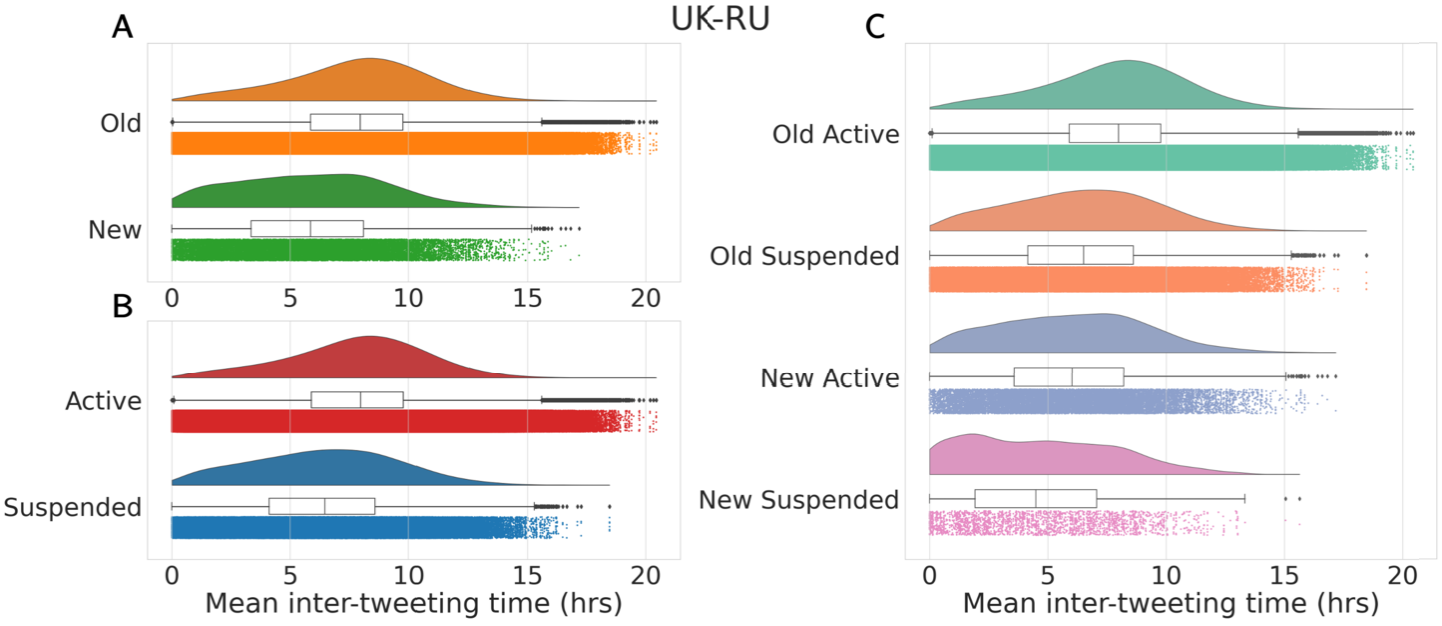
Distribution of the mean inter-tweeting time (in hours) for *Active* versus *Suspended* accounts **(A)**, *Old* versus *New* users **(B)** and all four classes **(C)**, in UK-RU. We only consider users that shared at least 10 tweets. Median values in **(A)** are 7.9 hours for *Active* and 6.4 hours for *Suspended*. Median values in **(B)** are 7.9 hours for *Old* and 5.8 hours for *New*. Median values in **(C)** are 7.9 hours for *Old Active*, 6.5 hours for *Old Suspended*, 6 hours for *New Active* and 4.4 hours for *New Suspended*. In each panel, distributions are statistically different according to two-sided Mann-Whitney tests ($p < 0.001$)

**Figure 11:**
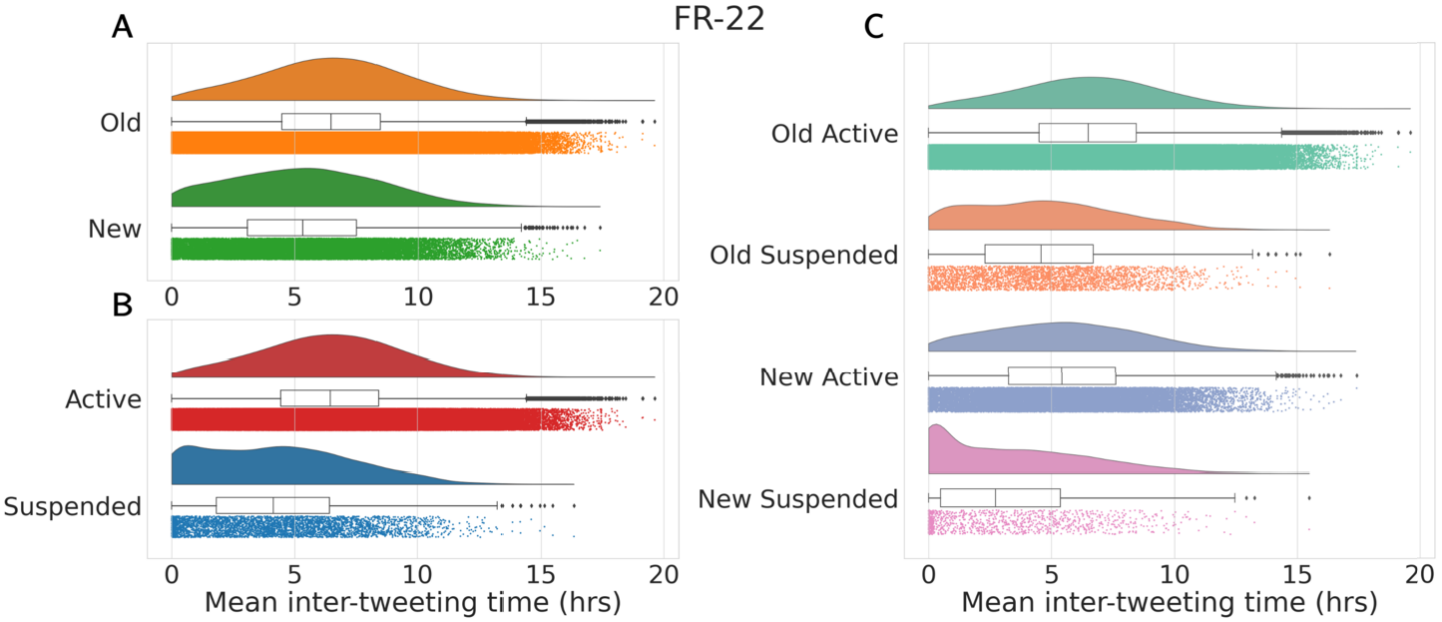
Distribution of the mean inter-tweeting time (in hours) for *Active* versus *Suspended* accounts **(A)**, *Old* versus *New* users **(B)** and all four classes **(C)**, in FR-22. We only consider users that shared at least 10 tweets. Median values in **(A)** are 6.46 hours for *Active* and 4.13 hours for *Suspended*. Median values in **(B)** are 6.49 hours for *Old* and 5.33 hours for *New*. Median values in **(C)** are 6.51 hours for *Old Active*, 4.59 hours for *Old Suspended*, 5.43 hours for *New Active* and 2.72 hours for *New Suspended*. In each panel, distributions are statistically different according to two-sided Mann-Whitney tests ($p < 0.001$)

Lastly, we analyzed the inter-tweeting time of different classes of users, considering only users with at least 10 tweets. As shown in Figs. 10 and 11, we first observe that *Suspended* accounts tweet with significantly higher frequency compared to *Active* users (panel A). Also, *New* users in general exhibit a much higher level of hyper-activity compared to other classes (panels B and C). Similar considerations hold for both datasets, and are in accordance with the past literature [28, 45].

### Patterns of interactions of suspended accounts (RQ2b)

Next, we analyze the interaction patterns enacted by *Suspended* and *Active* accounts, by considering replies, retweets, and quotes. Given class imbalance, most of the interactions specifically involve *Active* users, namely over 190 millions and 36 millions, respectively for UK-RU and FR-22. Conversely, just hundreds of thousands of interactions involve solely *Suspended* accounts. We thus normalize the number of interactions by source and target before searching for patterns, i.e., we respectively divide the number of interactions between two groups by the total amount of interactions generated (source) or received (target). Moreover, we employ a null model to statistically assess whether the amount of interactions taking place between different classes of users is larger or smaller than expected in a random ensemble obtained by assigning to users a random class label 100 times.

**Figure 12:**
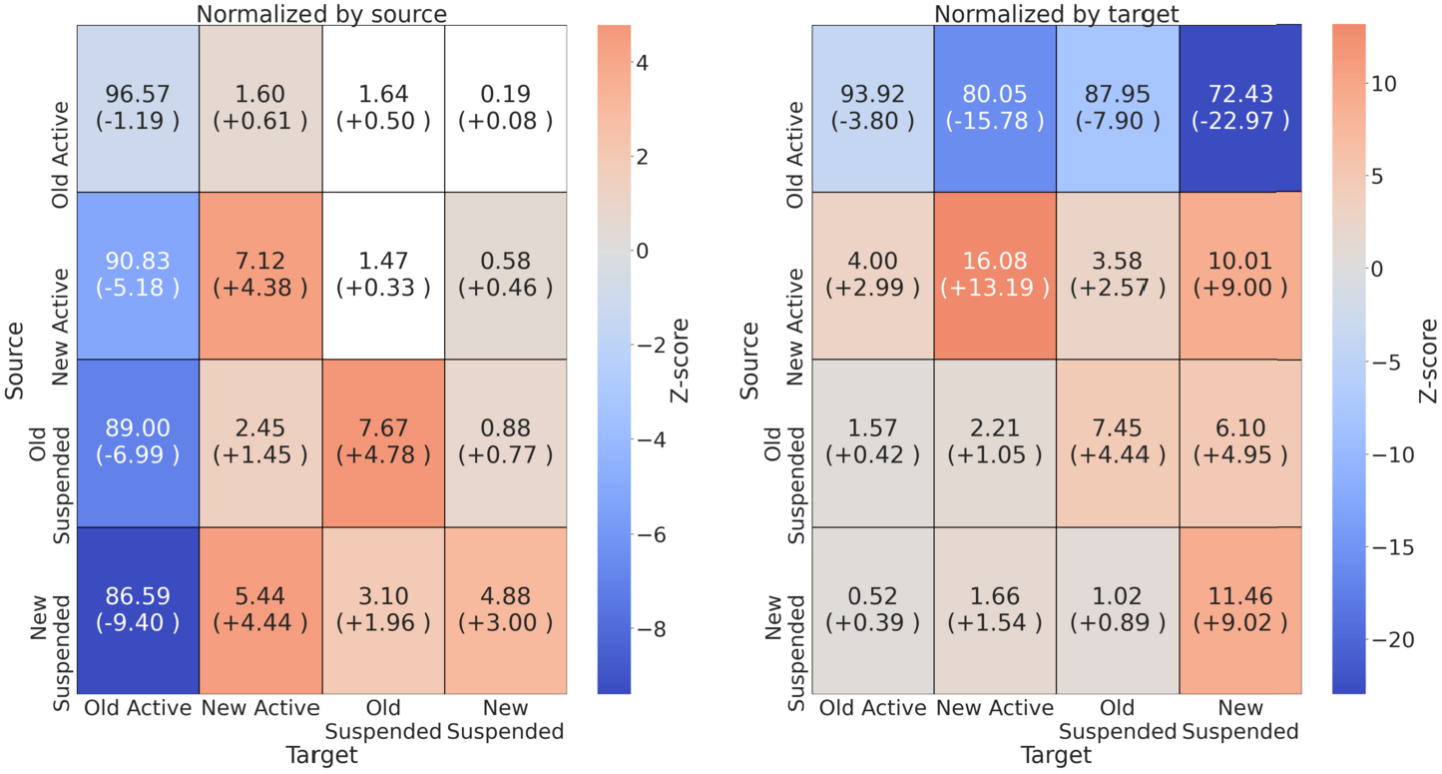
Heatmap of the observed amount of interactions normalized by source **(left)** and target **(right)** occurring between different classes of users and, in brackets, the difference with the expected value obtained through the null model, for the *UK-RU* dataset. Colors indicate Z-scores, and we only color cells with Z-score significant at $\alpha =0.05$. Cells are annotated with the normalized volume of interactions, and brackets report the difference w.r.t the value observed in the null model

**Figure 13:**
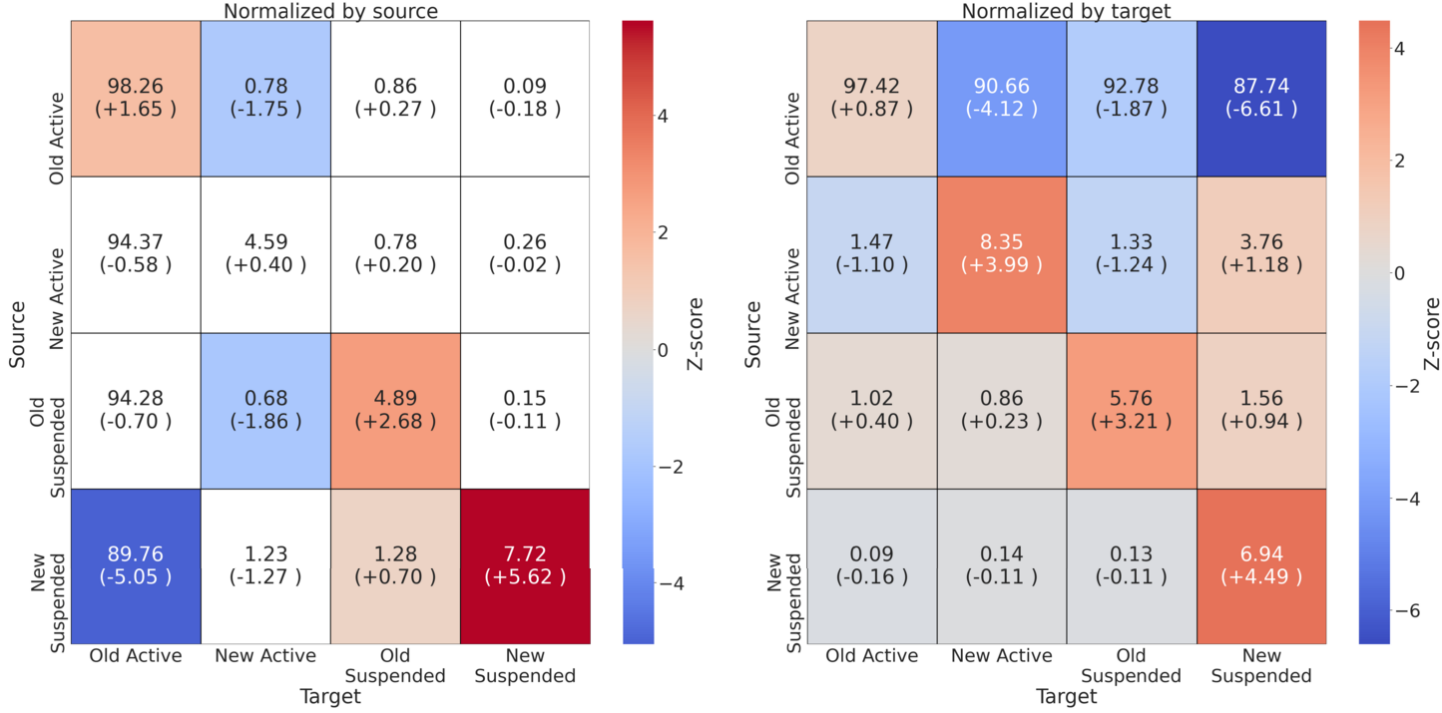
Heatmap of the observed amount of interactions normalized by source **(left)** and target **(right)** occurring between different classes of users and, in brackets, the difference with the expected value obtained through the null model, for the *FR-22* dataset. Colors indicate Z-scores, and we only color cells with Z-score significant at $\alpha =0.05$. Cells are annotated with the normalized volume of interactions, and brackets report the difference w.r.t the value observed in the null model

We show results for UK-RU in Fig. 12, where we provide a heatmap with statistically significant values. We can see that the amount of interactions originating from *Old Active* users is smaller than expected—both when normalized by source or target—whereas interactions between other classes of users are higher than expected. Figure 13 shows different results for FR-22: interactions normalized by source are mostly not significant, with a larger amount of interactions within *New Suspended* users than expected; interactions normalized by the target are smaller than expected when originating from *Old Active* users, and larger than expected for *New Active*, *Old Suspended* and *New Suspended* users when they interact with users from the same class.

We further looked at the interactions between groups over time. The time series of interactions from/to *Active* users simply reflects the trend shown in Fig. 1, in both datasets. We report a decreasing trend in the number of interactions from/to *Old Suspended* accounts over time in both datasets—with no specific patterns among different actions—most likely due to the fact that many were suspended during the period of observation, thus reducing the sample over time and consequently the number of interactions.

**Figure 14:**
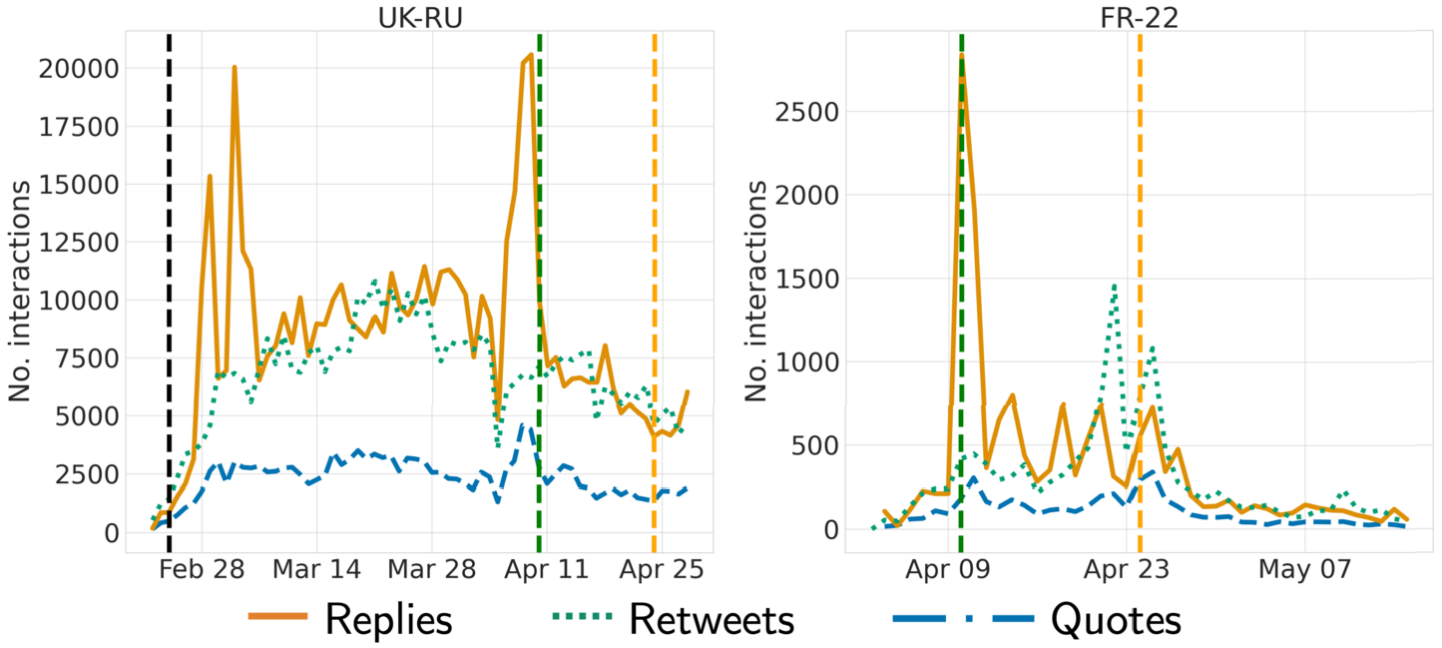
Number of interactions directed from New Suspended accounts to other accounts in UK-RU **(left)** and FR-22 **(right)**. Vertical lines indicate the invasion of Ukraine (black) and the two rounds of elections (green and light orange)

Focusing on interactions taking place among *New Suspended* accounts, as shown in Fig. 14, we notice some spikes in the number of replies (sent by these accounts) in both datasets, coherently with the behavior highlighted in the previous section; interestingly, these spikes are aligned with the peak of account creation (cf., Fig. 2). Additionally, we observe spikes of interactions among *New Suspended* accounts on specific days, in both datasets, which might indicate spam activity or other malicious behaviors in need of further investigation.

### Content characterization of suspended accounts (RQ3)

**Figure 15:**
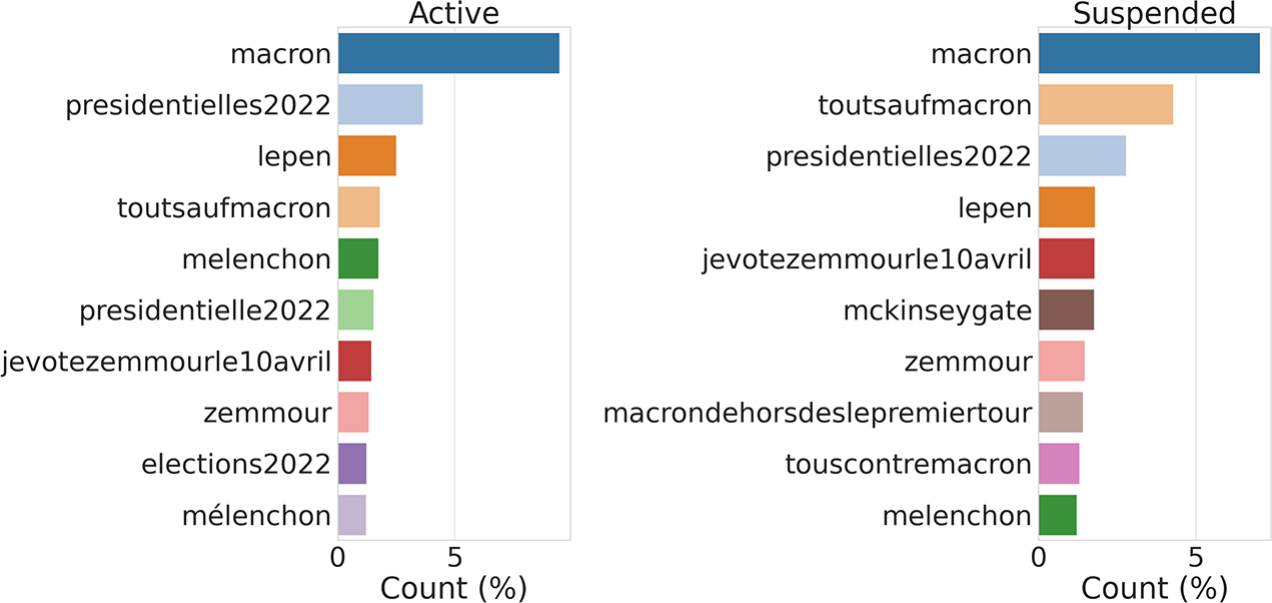
Top-10 most frequent hashtags shared by Suspended and Active users in FR-22

**Figure 16:**
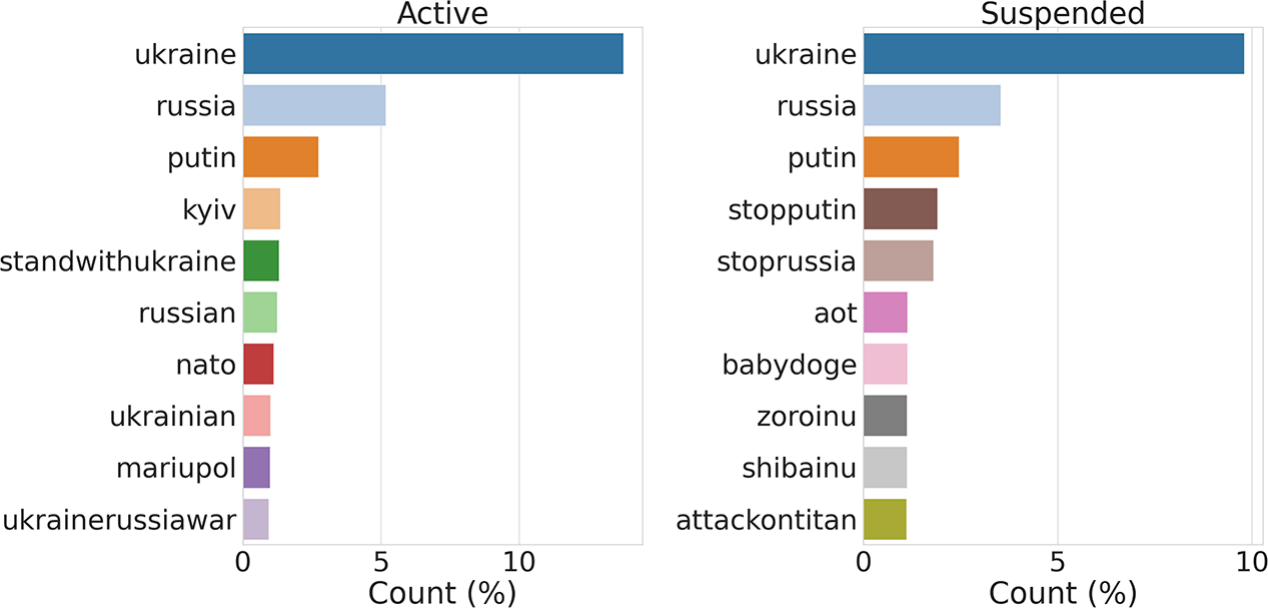
Top-10 most frequent hashtags shared by Suspended and Active users in UK-RU

We analyzed the content shared by *Suspended* and *Active* accounts by first extracting the Uniform Resource Locators (URL) and hashtags most shared during the period of analysis by each of the two macro-groups. We do not report relevant differences when looking at top web domains. For what concerns hashtags: In FR-22, we observe a slightly higher presence of inflammatory hashtags against Macron (e.g. #toutsaufmacron) and #touscontremacron) shared by *Suspended* accounts compared to *Active* ones (see Fig. 15, in line with previous research [31, 35, 63]; In UK-RU, we observe that Suspended users shared several hashtags related to Non-Fungible-Tokens (NFT) and cryptocurrency-related spam (e.g., #babydoge and #shibainu) see Fig. 16), in line with other recent studies [64–66].

Next, two annotators independently (and manually) labeled a random sample of 100 original tweets, replies, or quotes (we intentionally excluded retweets) shared by accounts in each class, namely *New Active*, *Old Active*, *New Suspended* and *Old Suspended* accounts. Annotators did not have information about the class of the user that shared each tweet (i.e., if they were active/suspended or new/old), and there were only 73 coding disagreements out of 800 tweets, which were discussed in order to reach an agreement on a single label. We referred to the labeling taxonomy introduced in [67], where authors considered the following categories: Offensive language, Abusive language, Hate speech, Aggressive behavior, Cyberbullying behavior, Spam, and Normal. We collapsed the first 5 categories into one macro-category (Harmful) and thus considered three classes for understanding the type of messages posted by suspended accounts: Harmful, Normal, and Spam.

**Figure 17:**
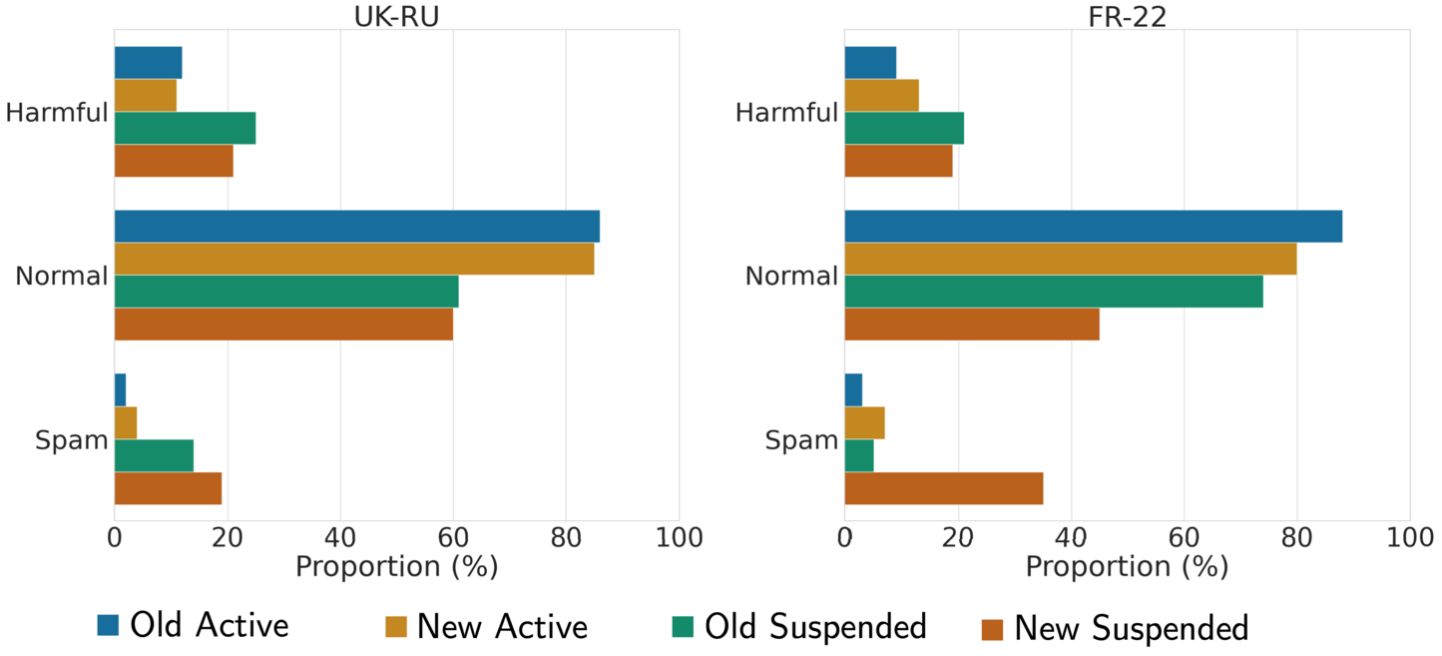
Proportion of tweets that were labeled as Harmful, Normal or Spam for each class of accounts for *UK-RU* **(left)** and *FR-22* **(right)**

We show the resulting proportion of tweets shared by different classes of users for each category (and dataset) in Fig. 17. We find similar results in both datasets: suspended users shared a larger amount of Harmful and Spam messages compared to active users; *New Suspended* users were particularly active in spamming campaigns in both datasets, and these findings are in line with previous work [27, 43, 44].

## Discussion

### Contributions

We studied the dynamics of account creation and suspension on Twitter during major geopolitical events such as the Russian-Ukraine conflict and the 2022 French Presidential election, showing results that are generalizable to different settings. Leveraging a large-scale dataset of 270M tweets in multiple languages shared by over 16M users, we uncovered peaks in the creation of accounts which in some cases were associated with specific events such as the election rounds. These included a large number of users that were later suspended for violating Twitter’s policies. We highlighted how Twitter tends to be more proactive towards recently created accounts compared to users with a longer lifespan. We analyzed in detail the behavioral features which characterize suspended accounts from legitimate active ones, showing an excessive usage of replies and toxic language by the former group as well as a higher level of activity. We studied the interactions between different classes of users, finding that abusive users were only successful at reciprocating with new legitimate ones throughout the period of observation, but not with the old accounts. Finally, by means of a qualitative analysis of a small sample of tweets, we estimated that suspended accounts frequently shared harmful and spam messages, which most likely lead to their deactivation.

### Limitations

There are a number of limitations in our study. Due to the 1% limit in Twitter’s Filter API [60], we were occasionally unable to capture the full volume of conversations related to the war in Ukraine, potentially missing peaks of account creation and activity; we did not incur in the same issue with the French Presidential election data collection. However, finding many similar results across the two datasets suggests that our data sample was not severely affected by this limitation. Our list of keywords might not include all the different spellings and transliterations of Ukrainian and Russian words, and our dataset might miss some relevant Twitter activity. As Twitter does not release details on the reasons behind the suspension nor the timestamp of the event, our proxy approach to detect suspension might be prone to error. It might also be the case that different moderation efforts have been applied in the two settings, and that policies are likely to change over time. We did not filter out automated accounts, i.e., social bots [68], and an investigation to relate bot behavior to suspension is left for future research, considering that a correlation between account suspension and increased bot likeness was already observed in other social and political discussions [69, 70]. Lastly, there are two sources of confounding/unobserved factors when studying the characteristics of users who get suspended. One comes from the scope of our analysis, as we only analyze tweets related to a given topic of conversation, e.g., the ongoing conflict or the election, and users might be engaging in other conversations that are not captured by our data; this would require to collect data from each user in a separate dedicated stream. The other source of confounding factors comes from the fact that many active users might exhibit malicious behaviors similar to suspended users, but have not been flagged or detected yet by the platform.

### Conclusions and future work

Our results show that social media platforms are particularly exposed to digital harm and online manipulation during events that captivate the public discourse on a large scale, when the volume of conversations and user engagement increases rapidly. Our work contributes to the extant literature on the behavioral dynamics of users who pollute social media platforms, but numerous questions still remain open in relation to the behavior of malicious actors and their influence on online conversations. This is particularly relevant given existing limitations to accessing data from social platforms, which do not transparently disclose how they detect and remove harmful content and accounts.

On the one hand, the spread of false information, hate speech, and other shenanigans on online social media are detrimental to the democratic process. On the other hand, platform interventions might be perceived as posing threats to freedom of speech and, as one unintended consequence, deplatforming can cause migration to other fringe communities, which are harder to map and study, leading to an increase in extremism of online activity. Our results call for more transparent collaborations between academics, platforms, and regulators in order to devise effective strategies to cope with online harm and manipulation in a timely manner, especially during major events that involve massive audiences.

Future work might build upon our findings to design algorithms to automatically predict users that will be violating platforms’ terms, as well as spotlighting users that behave maliciously but have not been suspended yet. Researchers could further investigate the presence of automated and (inauthentic) coordinated behavior [6, 7], which might involve users that get suspended, and they could perform a deeper investigation of the content shared by these users at scale. Finally, future research could consider multiple platforms simultaneously, and study the role of suspended users in other domains, such as medical or scientific topics.

### Ethical considerations

In the spirit of transparency and open research, we provide public access to the two datasets collected for this study. Both datasets consist of public posts collected via APIs that are accessible to the general public. To abide by Twitter’s terms of service we only release IDs of tweets. These can be used to retrieve the data analyzed in this paper, with the exception of posts that have been removed or made private by users, thus limiting reproducibility analyses. We note that we employed Twitter’s Compliance API instead of the status/lookup endpoint given its possibility to scale to millions of users, similar to previous work [51, 62, 71]. Despite the fact that it is not designed for such application, we deem its usage reasonable to hold platforms accountable for the integrity of online conversations during critical events such as global conflicts and political elections. At the time of this writing, we also acknowledge that access for researchers to Twitter’s API might be limited in light of the new policies, and this might hinder future usage of our resources. Following standard ethical guidelines, we did not attempt to identify or de-anonymize users, and we only report aggregate analyses. We acknowledge that malicious actors might exploit our results to better understand platforms’ moderation policies and devise strategies to avoid being detected and suspended, especially when conducting harmful campaigns during relevant global events.

Note: this project was approved by our institution’s IRB.

## Data Availability

We provide full access to IDs of tweets analyzed in our work, which can be retrieved using Twitter’s API. The datasets are available https://github.com/echen102/ukraine-russia and https://github.com/echen102/fr_election_2022.

## Footnotes

1 theconversation.com/deplatforming-online-extremists-reduces-their-followers-but-theres-a-price-188674.

2 developer.twitter.com/en/docs/twitter-api/v1.

3 github.com/echen102/ukraine-russia.

4 github.com/DocNow/hydrator.

5 twarc-project.readthedocs.io/en/latest.

6 github.com/echen102/fr-elections-2022.

7 twarc-project.readthedocs.io/en/latest/twarc2_en_us/#compliance-job.

8 help.twitter.com/en/managing-your-account/suspended-twitter-accounts.

## Abbreviations

API: Application Programming Interface
URL: Uniform Resource Locator
